# Alloimmune Neutropenia in a Neonate: Case Report and Review of Literature

**DOI:** 10.3390/antib11040063

**Published:** 2022-10-03

**Authors:** Arun Prasath, Alanna Grafius, Mona Bonanno, Steven Ambrusko, Jayasree Nair

**Affiliations:** Department of Pediatrics, Jacobs School of Medicine and Biomedical Sciences, University at Buffalo, Buffalo, NY 14203, USA

**Keywords:** neonatal neutropenia, anti-neutrophil antibodies, alloimmune neutropenia

## Abstract

Neonatal alloimmune neutropenia, variably referred to in the literature as NAIN, FNAIN or NIN, is a disorder of neutrophil destruction in newborns similar to better-known conditions such as hemolytic disease of the newborn and neonatal alloimmune thrombocytopenia (FNAIT). Infants affected by this self-limiting condition can present asymptomatically or have a wide range of symptoms, from skin manifestations and mucositis to severe infections such as sepsis and pneumonia. In our case, we report an otherwise asymptomatic term infant born with severe neutropenia to a mother affected by COVID-19 in the 3rd trimester. However, it is unclear if COVID-19 contributed to our patients’ neutropenia. Diagnostic testing eventually revealed the presence of anti-neutrophil antibodies, confirming the diagnosis of alloimmune neutropenia. The infant was conservatively managed with early discharge prior to resolution of neutropenia and close post-discharge follow up.

## 1. Introduction

Neutropenia is not unusual in the neonatal ICU but is usually seen in intra-uterine growth-restricted infants, extremely preterm infants and infants born to mothers with preeclampsia. Severe persistent neutropenia in an appropriate-for-gestational-age term infant is uncommon and suggests an unusual etiology, requiring extensive and often multidisciplinary evaluation to reach a diagnosis. Neonatal alloimmune neutropenia (variably termed as NAIN, ANN, and FNAIN) is one such condition that occurs due to the destruction of neonatal neutrophils as a result of transplacental transfer of anti-neutrophil antibodies against surface neutrophil antigens inherited by the infant from the paternal side [1,2]. This diagnosis must be suspected in an otherwise asymptomatic neonate with severe neutropenia. We report an infant with isolated severe neutropenia, with benign dermatological lesions. Maternal gestational hypertension prompted blood count testing, which revealed severe neutropenia.

## 2. Case

A Caucasian female infant was born at 38 weeks 1 day gestational age to a primigravida mother with negative routine screening for hepatitis B, HIV, rubella and syphilis. The pregnancy was complicated by dichorionic diamniotic twin gestation with fetal demise of twin at 11 weeks gestation, hyperemesis requiring PICC line, maternal SARS-CoV-2 infection at 33 weeks gestation, and gestational hypertension with onset at 37 weeks not requiring medication. The mother did not have any family history of autoimmune diseases.

The infant was born via scheduled Caesarian section due to fetal malposition with frank breech presentation. APGAR scores were 9 and 9 at 1 and 5 min respectively, and the well-appearing infant was transferred to the mother-baby unit. A diffuse rash was noted on the face and trunks, which appeared to be prominent facial vascular nevi around the eyelid, with multiple similar areas noted on the trunk and lower extremities. Blood counts sent by the pediatrician at 8 h of life revealed leukopenia and neutropenia. Infant was clinically asymptomatic with stable vital signs, good perfusion and no distress. Due to persistent neutropenia, the infant was evaluated for underlying infection. A blood culture was sent and empiric antibiotic therapy with ampicillin and gentamicin initiated.

## 3. Course

The infant was transferred to a tertiary care neonatal intensive care unit for further evaluation of severe neutropenia with skin findings. Serial blood counts are represented in Table 1. Besides the absolute neutrophil counts, other blood count parameters were mostly within normal range.

Pediatric hematology, as well as dermatology evaluations, were initiated. Hematology recommended following serial blood counts as long as the infant remained clinically stable. Skin lesions were thought to be vascular in nature; therefore, the infant had significant imaging to rule out further vascular abnormalities. Abdominal ultrasound, head ultrasound, and MRI brain/orbits were all negative. Eyelid lesion was determined to be consistent with telangiectasia and infant was diagnosed with cutis marmorata telangiectasia congenita, a benign skin condition unrelated to the neutropenia. Infectious screens sent ruled out common viral etiologies such as herpes simplex virus as well as Toxoplasma (IgG, IgM negative). SARS-CoV-2 screen was also negative. Antibiotics were discontinued after 72 h once the blood culture was negative. As alloimmune neutropenia was considered, anti-neutrophil antibodies on the infant were tested. Infant was discharged home at 9 days of age once the absolute neutrophil count (ANC) had improved from 0 to 366. After discharge, the anti-neutrophil antibody test was positive, confirming the diagnosis of alloimmune neutropenia. The baby was followed in outpatient pediatric hematology clinic and had steadily rising neutrophil counts to over 2500 on a subsequent visit at 2 weeks after discharge. HNA genotyping to identify the antigen was not performed.

## 4. Review of the Literature: Neonatal Alloimmune Neutropenia

Eight percent of neonates in a NICU can have neutropenia [1,2]. Common causes include bacterial or viral infection, congenital neutropenia, and immune-mediated neutropenia. While post-infectious neutropenia is relatively common, the presence of isolated severe neutropenia has recently been described in children with COVID-19/ SARS-CoV-2 infection [3,4]. Neonatal neutropenia may also be associated with multisystem inflammatory syndrome in children (MIS-C) [5]. However, the infant we report tested negative for SARS-CoV-2 and did not have any symptoms of infection. Neonatal neutropenia as a consequence of maternal COVID-19 infection during pregnancy is not commonly described [6].

Of immune neutropenias, neutropenia may present in a neonate from maternal autoantibodies or allosensitization against fetal neutrophils; true autoimmune neutropenia in neonates is exceedingly rare. Neonatal alloimmune neutropenia is seen with a maternal history of autoimmune disease, when maternal neutrophil autoantibodies are passively transferred to the fetus [7]. In the absence of maternal autoimmune disease or maternal neutropenia, alloimmune neutropenia should be suspected.

### 4.1. Incidence and Pathogenesis

The mechanism of alloimmune neutropenia is similar to that of hemolytic disease of the fetus and newborn (HDFN) and fetal/neonatal alloimmune thrombocytopenia. The IgG antibodies transferred can cause neonatal neutropenia lasting for a period of weeks to months, which can be severe. The reported incidence of alloimmune neutropenia in the literature varies from 0.1–0.2% [8,9], though a higher percentage report the presence of alloimmunization without significant neutropenia [10]. A total of 5–40% of NAIN are reported with first pregnancies, implying that maternal immunization/exposure can take place prior to or during that pregnancy [11,12]. Identified neutrophil antigens are classified based on the human neutrophil antigen (HNA) system and include 11 HNAs within five different HNA systems: four HNA-1 alleles, HNA-2, two HNA-3, two HNA-4 alleles, and two HNA-5 alleles [13], with HNA-1 being most commonly identified.

### 4.2. Clinical Features and Diagnosis

Alloimmune neutropenia was first described in a family with siblings presenting with pneumonia, sepsis and omphalitis [14]. Various infections are often noted in this condition. In a cohort of 35 patients with NAIN, 60% had infections within the first 2 weeks after birth which led to identification of neutropenia, while in 40%, neutropenia was noted incidentally on blood counts done for other reasons [12]. Skin infections have also been identified as a presenting symptom in some infants with this condition. Our term infant was asymptomatic except for a rash, which prompted blood counts and identified neutropenia. Some studies have reported a high incidence of miscarriage associated with this condition (6–50%) [8,11,12]. Interestingly, our infant was the product of a twin pregnancy with early fetal demise of the twin. Due to severe neutropenia, this condition can be potentially fatal if serious infection occurs and management is not initiated early. The mortality rate in this condition could be about 5% [15]. NAIN is self-limiting, resolving within a few weeks to 6 months, as the levels of acquired maternal antibody diminish [16].

Diagnosis is confirmed by demonstration of antineutrophil antibodies in the baby, as in our infant, but could also be performed in the mother. HNA genotyping of the mother and father confirms the diagnosis. HNA genotyping was not performed in the parents of our patient, though parents were advised testing. Since future pregnancies could be affected, counseling of families is important.

### 4.3. Management

Management depends on presentation and clinical symptoms. In infants presenting with an infection, therapy with appropriate antibiotics, as well as treatment with granulocyte colony stimulating factor (GCSF), should be considered [17]. While the effectiveness of prophylactic GCSF remains in question, its use in infants with significant infections produces a robust response with a rise in neutrophil counts within 1–3 days [18] and is recommended. GCSF potentially stimulates the release of neutrophils from bone marrow, thus inducing myeloid proliferation and increasing circulating neutrophil numbers [19]. Another proposed mechanism is that GCSF downregulates antigen expression, thus making neutrophils less vulnerable to circulating antibodies [20]. Rare resistance to GCSF has been reported, especially associated with HNA 2a [21]. Prophylactic antibiotics are almost always initiated in infants with severe neutropenia, however there is no data to suggest any benefit to continuing an antibiotic course once infection has been ruled out. Our infant received antibiotics for 72 h, but they were discontinued as the infant remained clinically stable and blood culture showed no growth. GCSF was considered in our patient but not initiated in the absence of clinical symptom or vital sign instability suggestive for infection.

Intravenous immunoglobulin (IVIG) use has also been reported in this condition [22], however most infants show a good response to antibiotics and/or GCSF without IVIG. The effectiveness of IVIG therapy for sepsis has been questioned [23], but it remains an option for second-line treatment. Systemic steroid use in this condition has also been reported [14,15].

## 5. Conclusions

Neonatal alloimmune neutropenia should be considered in infants presenting with severe neutropenia, either incidentally or with concurrent symptomatology soon after birth. As we demonstrated in our case, conservative management of asymptomatic infants is possible despite persistent neutropenia. Infants require close follow-up and detailed anticipatory guidance to ensure resolution, which can take up to 6 months.

## Figures and Tables

**Table 1:** Serial blood counts in infant during the hospital stay, showing a nadir between days 4–7, followed by recovery.

Age at Which Test Done	WBC Count (Ref: 5.0–30.0) (×10^3^)	Absolute Neutrophil Count (Ref: 1500–21,000) (cells/microL)	Hematocrit (Ref: 40–61) (%)	Platelet Count (Ref: 150–450) (×10^3^)
8 h	7.3	730	44.6	223
16 h	6.6	330	40.8	134
36 h	4.9	343	39.4	207
Day 3	6.1	61	41.5	219
Day 4	6.0	0	40.9	206
Day 5	5.5	275	40.5	243
Day 6	6.0	120	36.8	243
Day 7	6.7	0	37.1	267
Day 8	5.3	159	36.3	152
Day 9	6.1	366	35.8	294
Day 16 @@@@(outpatient)	9.4	2540	36.6	

## Data Availability

Not applicable.
